# Naturally-occurring, dually-functional fusions between restriction endonucleases and regulatory proteins

**DOI:** 10.1186/1471-2148-13-218

**Published:** 2013-10-02

**Authors:** Jixiao Liang, Robert M Blumenthal

**Affiliations:** 1 Department of Medical Microbiology & Immunology, College of Medicine and Life Sciences, University of Toledo, 3100 Transverse Drive, Toledo, OH 43614, USA; 2 Program in Bioinformatics, University of Toledo, 3100 Transverse Drive, Toledo, OH 43614, USA

**Keywords:** Restriction-modification systems, Restriction endonuclease, Gene regulation, Fused genes, C protein, Regulatory evolution

## Abstract

**Background:**

Restriction-modification (RM) systems appear to play key roles in modulating gene flow among bacteria and archaea. Because the restriction endonuclease (REase) is potentially lethal to unmethylated new host cells, regulation to ensure pre-expression of the protective DNA methyltransferase (MTase) is essential to the spread of RM genes. This is particularly true for Type IIP RM systems, in which the REase and MTase are separate, independently-active proteins. A substantial subset of Type IIP RM systems are controlled by an activator-repressor called C protein. In these systems, C controls the promoter for its own gene, and for the downstream REase gene that lacks its own promoter. Thus MTase is expressed immediately after the RM genes enter a new cell, while expression of REase is delayed until sufficient C protein accumulates. To study the variation in and evolution of this regulatory mechanism, we searched for RM systems closely related to the well-studied C protein-dependent PvuII RM system. Unexpectedly, among those found were several in which the C protein and REase genes were fused.

**Results:**

The gene for CR.NsoJS138I fusion protein (*nsoJS138ICR*, from the bacterium *Niabella soli*) was cloned, and the fusion protein produced and partially purified. Western blots provided no evidence that, under the conditions tested, anything other than full-length fusion protein is produced. This protein had REase activity *in vitro* and, as expected from the sequence similarity, its specificity was indistinguishable from that for PvuII REase, though the optimal reaction conditions were different. Furthermore, the fusion was active as a C protein, as revealed by *in vivo* activation of a *lacZ* reporter fusion to the promoter region for the *nsoJS138ICR* gene.

**Conclusions:**

Fusions between C proteins and REases have not previously been characterized, though other fusions have (such as between REases and MTases). These results reinforce the evidence for impressive modularity among RM system proteins, and raise important questions about the implications of the C-REase fusions on expression kinetics of these RM systems.

## Background

**Figure 1:**
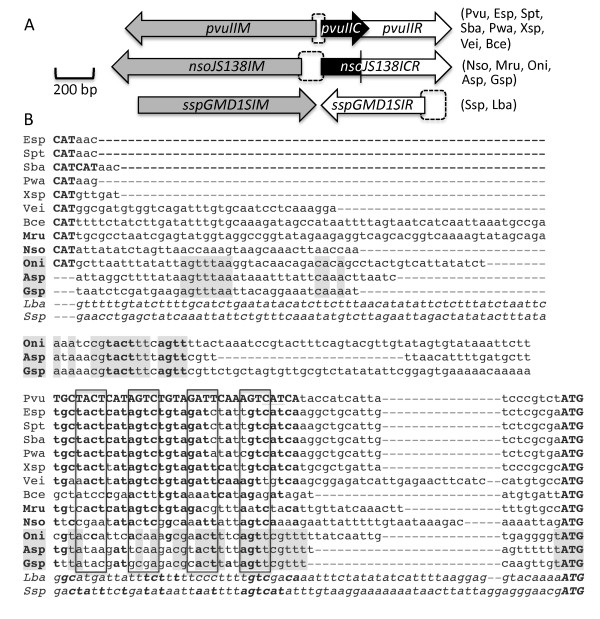
**Genetic maps and putative regulatory DNA sequences for RM systems containing R.PvuII orthologs. A**. Three classes of RM systems containing R.PvuII orthologs. Top is PvuII itself, and systems at the right share its basic layout. In the middle is NsoJ138I, exemplifying systems with a fused C-REase protein. The systems at right share this layout, though in Asp and Gsp the MTase gene is in the opposite orientation. At the bottom is a streptococcal system (“Ssp”) that lacks a C gene, as does a system from Lachnospiraceae (“Lba”). **B**. Putative regulatory regions. The ATG at bottom right is the initiation codon for the C gene (except for Lba and Ssp, which have no C gene so the REase initiator is shown). The names of systems with fused C-REase genes are in bold; the subset from cyanobacteria are shaded (sequences shared by these three are also shaded). Most systems have the MTase gene divergently oriented from the C gene, and the initiator reverse complement is indicated in bold (CAT); the MTase initiator for Pvu is very close to that for the C gene (overlapping the downstream-most C box, and underlined). In the Asp, Gsp, Ssp and Lba systems the MTase and C (REase) are convergent. Rectangles indicate symmetry elements from C boxes (based on PvuII), with matches in other systems indicated in bold. Lba and Ssp, which lack a C gene and (presumably) C boxes, are shown for comparison. Species sources are: Pvu (*Proteus vulgaris*), Esp (*Escherichia* spp. TW09308), Spt (*Salmonella enterica* Paratyphi A), Sba (*Salmonella enterica* Bareilly), Pwa (*Pectobacterium wasabei*), Xsp (*Xanthomonas* spp.), Vei (*Verminephrobacter eiseniae*), Bce (*Bacteroides cellulosilyticus*), Mru (*Meiothermus ruber*), Nso (*Niabella soli*), Oni (*Oscillatoria nigro-viridis*), Asp (*Anabaena* spp.), Gsp (*Gloeocapsa* spp.), Lba (*Lachnospiraceae bacterium*), and Ssp (*Streptococcus* spp. GMD1S). GenBank accession numbers are in Methods.

Restriction-modification (RM) systems appear to play key roles in modulating gene flow among bacteria and archaea. This includes not only defense against bacteriophages [1,2], but also negative and positive modulation of interspecies gene transfers [3,4]. Because the restriction endonuclease (REase) is potentially lethal to unmethylated new host cells [5,6], regulation to ensure pre-expression of the protective DNA methyltransferase (MTase) is essential to the spread of RM genes. This is particularly true for Type IIP RM systems, in which the REase and MTase are separate, independently-active proteins [7]. A substantial subset of Type IIP RM systems are controlled by an activator-repressor called C protein [8,9]. In these systems, C controls the promoter for its own gene, and for the downstream REase gene that lacks its own promoter [10] (Figure 1A). In tested C-protein-dependent RM systems, including PvuII, the C protein both activates and represses this promoter [9,11-14]. In some cases, this process has been studied structurally [15-17] and by mathematical modelling [18,19]. The C protein operators, called C boxes, have recognizable sequences with symmetrical elements upstream of the C ORFs [8,10,20,21]. Thus MTase is expressed immediately after the RM genes enter a new cell, while expression of REase is delayed until sufficient C protein accumulates [22].

To study the variation in and evolution of this regulatory mechanism, we searched for RM systems closely related to the well-studied C protein-dependent PvuII RM system. The PvuII system was discovered and cloned nearly three decades ago [23,24], yielded the discovery of C proteins [9], and has been subject to structural [25-27], evolutionary [28], and detailed regulatory studies [10,19,21,22,29,30]. PvuII thus represented a good starting point for studies on the evolution of the C protein-dependent regulatory mechanism.

Unexpectedly, among the PvuII-orthologous RM systems that we found were several in which the C protein and REase genes were translationally fused. One of these, selected for further study, was the NsoJS138I RM system from the bacterium *Niabella soli*. We report here that the NsoJS138I fused protein is produced, and is functional for both C protein and REase activities.

## Results

### Identification of RM systems containing genes orthologous to *pvuIIR*

We began our studies on evolution and variation of RM regulation by identifying RM systems that contained genes orthologous to the PvuII REase gene, *pvuIIR*. We have identified such RM systems in the past [28], and the use of the REase gene for such searches yielded the best results, in terms of returning the most clearly orthologous systems. This is because the C proteins have substantial sequence identities even when coming from unrelated RM systems [8,9], and the MTases similarly have well-conserved sequence motifs [31,32]. However, sequence and structural similarities among REases are quite limited [33-35].

We used the amino acid sequence of R.PvuII (gi 135242) as the search seed (initial query), and examined all available bacterial and archaeal genome sequences (complete and shotgun) using the program TBlastN [36]. The aligned REase sequences are shown in Figure S1 (note: all supplementary figures are in Additional file 1), and an unrooted tree indicates their relatedness in Additional file 1: Figure S2. The regulatory regions from these systems are shown in Figure 1B. Of ten RM systems identified (including two from a previous study [28], and excluding identical systems), nine were like PvuII in that they also contained a C protein gene and had the MTase gene divergently oriented from that of C and the REase (Figure 1A).

Interestingly, we found a PvuII-orthologous system that lacked the C protein gene altogether, in the Gram-positive Clostridium-related family Lachnospiraceae. In this RM system, the MTase and REase genes are convergent rather than divergent, though there are numerous examples of C-regulated RM systems with convergent orientations [9,37]. Not surprisingly, there is no significant sign of C-protein binding sites (C boxes) in the Lachnospira sequence (Figure 1B). Using this REase aa sequence as a search seed revealed a closely-related RM system lacking a C gene in the Gram-positive genus Streptococcus (Figure 1A). This is also shown in Figure 1B, and also lacks obvious C boxes. Its regulatory region exhibits no significant similarity to the one upstream of the Lachnospira REase gene (bottom lines in Figure 1B), even though the Lachnospira and Streptococcus REase amino acid sequences are closely related (Additional file 1: Figure S2). It could be informative, with respect to our understanding of how the regulation of these RM systems evolves, to know how these two systems are controlled and whether they use the same mechanisms.

**Figure 2:**
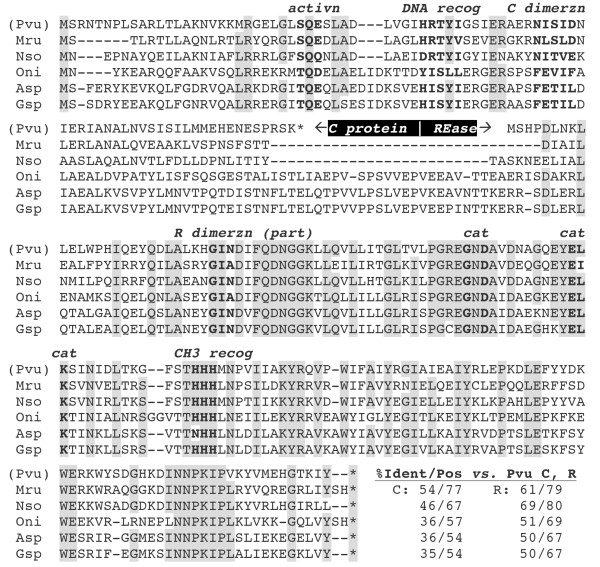
**Alignment of CR fusion proteins orthologous to C.PvuII and R.PvuII.** The PvuII system (top line) is unfused, and shown for comparison. Fully conserved positions or those conserved in 5/6 systems are shaded, and known functional regions (in PvuII) are in bold font (transcriptional activation, recognition helix of the helix-turn-helix motif, dimerization interfaces, Mg^++^-coordinating residues for REase catalysis (cat), and recognition of the methylated (CH3) base to prevent cleavage. Species sources are as described in the Figure 1 legend. At the lower right, the numbers indicate % amino acid identity (“Ident”) or similarity (“Pos”) for each RM system C or REase (“R”) portion to the corresponding PvuII polypeptide.

To our surprise we also found that, in two of the other PvuII-orthologous RM systems, the C protein and REase genes are translationally fused (Figure 1A). Furthermore, using these fused genes as search seeds, we identified three additional RM systems. The set of five R.PvuII-orthologous fused genes, along with the two unfused PvuII proteins for comparison, is shown in Figure 2. The C-orthologous portions of these five proteins range from 35-54% identity to C.PvuII (Figure 2 lower right). The REase portions of C-REase fusions range from 50-69% identity to R.PvuII, and are not all phylogenetically clustered, suggesting that fusion may have occurred more than once (Additional file 1: Figure S2). Specifically, the three cyanobacterial REase fusions very probably occurred separately from the Mru and Nso fusions, which in turn may or may not have been independent from one another.

These fused systems come from diverse bacteria: three are from genera in the phylum Cyanobacteria (Anabaena, Gloeocapsa and Oscillatoria), one is from the phylum Deinococcus-Thermus (Meiothermus), and one from the phylum Bacteroidetes (Niabella). The three cyanobacterial fusion proteins have a ~25 aa linker region between the C protein and REase regions, that is not present in the Meiothermus or Niabella fusions; among the conserved positions present in all three of the cyanobacterial linker regions are two Glu, three Thr, and three Pro. The three cyanobacterial fused systems also share features in their regulatory regions, including a putative C box well upstream of the usual position (bold text in shaded region, middle rows of Figure 1B). It is possible that this increased C box spacing is needed to accommodate DNA complexes with the fused proteins.

The RM database REBASE [38] includes regularly-updated automated searches for REases among DNA sequences. One of the fusions we found was originally noted in REBASE (*M. ruber*, Mru1279I, 10-Mar-2010/26-May-2013), and the rest were detected while this work was in progress but not notated as involving C-REase fusions (*A. species*, Asp90I, 17-Nov-2012; *G. species*, Gsp7428I, 23-Dec-2012; *N. soli*, NsoJS138I, 10-Apr-2013; *O. nigro-viridis*, Oni7112I, 20-Dec-2012). We have uniformly adopted the REBASE nomenclature for these RM systems.

### Isolation of genes for fused RM systems and their REase activity

The central question regarding these C-REase fusions is whether or not they are active. There are numerous examples of RM systems, identified through sequence comparisons, that do not produce catalytically active proteins [28,39]. We focused on two of the fused RM systems, isolating the *Meiothermus ruber* Mru1279I genes by amplification from genomic DNA (not shown), and having the *Niabella soli* NsoJS138I genes synthesized. We were unable to detect REase or MTase activity from the sequence-confirmed *M. ruber* clones (not shown), possibly due to poor expression in *E. coli* and/or improper folding of the protein at the lower *E. coli* growth temperature (37°C), though cell extracts were tested at the optimum for *M. ruber* growth (60°C) [40].

**Figure 3:**
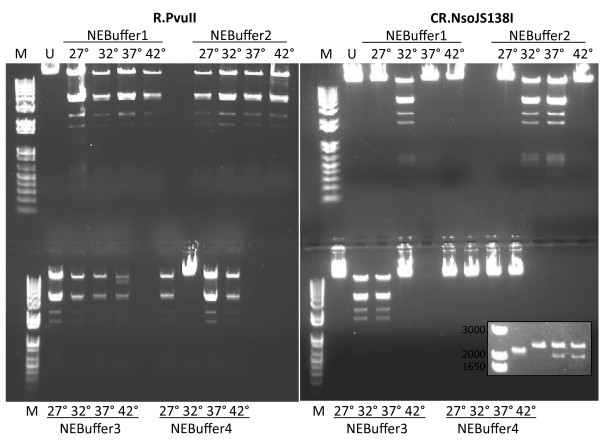
**Assessment of REase activity in CR.NsoJS138I.** A comparison was made between commercial R.PvuII and CR.NsoJS138I. Enzymes were incubated for 1h with DNA from bacteriophage λ. Four different reaction buffers were used, and in each buffer four temperatures were used. Reactions were resolved on agarose-TBE gels containing ethidium bromide (see Methods for details). **Inset:** Left-to-right: markers, uncut pUC19, pUC19 cut with CR.NsoJS138I or PvuII at 37°, pUC19 cut with CR.NsoJS138I at 30°.

In contrast, extracts from *E. coli* cultures carrying the *N. soli* genes gave obvious REase activity that indicated a specificity indistinguishable from that of PvuII REase. This was expected, given the sequence similarity (Figure 2). However, the NsoJS138I C-REase fusion exhibited much more stringent activity requirements than R.PvuII when they were tested at four temperatures in each of four buffers (Figures 3, Additional file 1: Figures S3, and S4). Specifically, R.PvuII was active in 15/16 tested conditions, while the fusion was active in 5/16. In particular, NsoJS138I was inactive at 27 and 42°C in all tested buffers, while PvuII was active in all buffers at those two temperatures. NsoJS138I was active in three buffers at 32° and two buffers at 37°C (Additional file 1: Figures S3 and S4), and serial dilution indicated that, at 32°C, NsoJS138I was most active in NEBuffer 3 (Additional file 1: Figure S4). These experiments used 10 u of PvuII from a commercial supplier; this is equivalent to ~20 ng of PvuII REase protein [41]. In comparison, 2.4 μg of NsoJS138ICR protein was used (~ 120× as much). Differences from R.PvuII could be due to the presence of the fused C portion at the amino ends of each subunit, to the sequence differences between the PvuII and NsoJS138I REase portions (Figure 2), or a combination of the two factors. The C-terminal His tag might also play a role, though it has little effect on R.PvuII.

### Production of CR.NsoJ138I as a fusion protein

**Figure 4:**
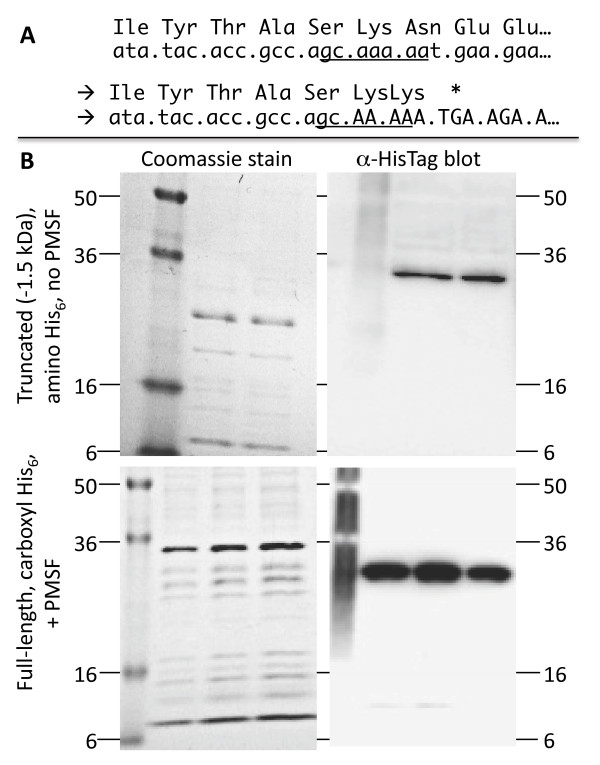
**Test of CR fusion protein production. (A)** The sequence spanning the C-REase junction has properties that might result in production of some free C protein. GCAAAAA has been associated with −1 ribosomal frameshifts (see text for references), and this would result here in termination at a nearby TGA triplet. **(B)** Production of NsoJS138I C-REase fusion protein, with an amino-terminal (upper) or carboxyl-terminal (lower) His_6_ tag, was induced using a T7 RNA polymerase-dependent promoter (see Methods). The upper panels show the results from clones having a small carboxyl-terminal deletion (done in case the REase activity proved to be toxic), while the lower panels show full-length clones. Centrifugally-clarified whole-cell extracts were passed over affinity columns to purify the His-tagged polypeptides, and resolved on duplicate 10-20% gradient acrylamide SDS gels. For the lower panels, the extracts were prepared in the presence of protease inhibitors. One gel of each pair was stained (left), the other was electroblotted and probed with anti-His-tag antiserum (see Methods). Loaded amounts of protein per lane were 2.0 μg (upper), and 3.4, 6.8 and 5.1 μg (lower, left to right).

The sequence of the NsoJ138I C-REase clearly indicates that a single fused polypeptide should be produced. However, it is possible that translational frameshifting [42] could result in the production of free C protein (as that portion is amino-proximal to the REase portion), or that proteolytic processing could result in both free C protein and free REase. In particular, the translational frameshifting is suggested by two features of the DNA sequence in the junction region (Figure 4A): one is a short sequence that has been associated with −1 translational frameshifts [43], and the other is a nearby stop codon in the −1 reading frame.

To test for these possibilities, we added a His_6_ tag to the amino or carboxyl end of the fusion protein, expressed the tagged proteins from a strong inducible promoter, partially purified cell extracts on affinity columns, and resolved the column eluates on SDS-polyacrylamide gels. Figure 4B shows the Coomassie stained gels next to western blots probed with anti-His_6_ antiserum, while Additional file 1: Figure S5 shows the amino-tagged protein isolated in the presence of protease inhibitor PMSF. Translational frameshifting would result in a ~9 kDa polypeptide in the extracts with amino-tagged fusion (the carboxyl-tagged fusion would only yield smaller protein in the case of proteolytic cleavage), and we see no evidence for that product. We cannot rule out the possibility that frameshifting occurs in the native host (*N. soli*), or in *E. coli* under different growth conditions. Nevertheless, the protein preparation used for the assays shown in Figure 3 was partially purified via a His_6_ affinity tag at the carboxyl end, and together with the results shown in the lower right panel of Figure 4B strongly suggest that the intact fusion protein is catalytically active.

### *In vivo* test of CR.NsoJ138I for C protein activity

**Figure 5:**
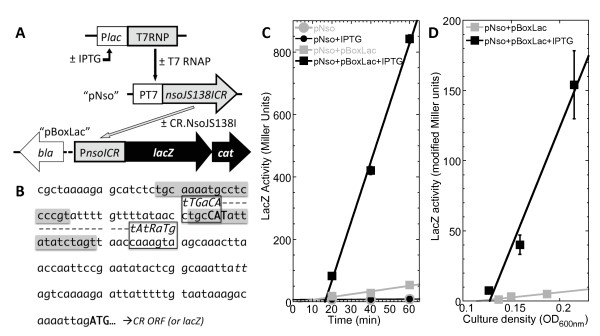
**Assessment of C activity in CR.NsoJS138I. A**. Schematic design of experiment. Top line indicates IPTG-inducible gene for T7 RNA polymerase in the host strain’s chromosome, middle indicates a plasmid (pJL200, called pNso for the figure) that carries the gene for CR.NsoJS138I linked to a T7 promoter, and the bottom indicates a plasmid (pJL300, called pBox-Lac for the figure) that carries the putative promoter and C box region from NsoJS138I linked to a promoterless gene for *lacZ* (β-galactosidase). **B**. Sequence of the putative promoter and C box region from NsoJS138I, showing the candidate C boxes (shaded) and promoter elements (−35 and −10 hexamers). This 161 nt sequence is what was included in pJL300 (pBoxLac). **C**. Timecourse of LacZ induction. Growing triplicate cultures of cells containing the indicated plasmids were treated at time = 0 with the inducer IPTG (which in these cells controls the gene for T7 RNA polymerase), and matched control cultures received no IPTG. LacZ activity was measured over time. The symbols indicate means of the triplicate cultures; standard errors are shown but mostly obscured by the symbols. **D**. Steady-state expression of *lacZ*. Triplicate cultures containing the plasmids indicated were grown for at least 10 generations in the presence or absence of IPTG, and LacZ activity was measured. In this case, activity is plotted *vs.* culture density, and so modified Miller units are used (in which the culture density term has been removed). Cultures approximating steady-state growth should give good linear fits, the slopes of which accurately measure relative expression levels. The symbols indicate means of the triplicate cultures; standard errors are shown but are in some cases obscured by the symbols.

Based on comparison to PvuII and other previously-studied C-dependent RM systems, we identified candidate C boxes upstream of the C-REase fusions, including NsoJ138I (Figure 1B). We also examined this region of the NsoJ138I sequence for putative bacterial promoters [44-46], with a candidate (boxed in Figure 5B) selected based on both its sequence and its position relative to the putative C boxes. A 161 bp sequence, including the putative C boxes and promoter (Figure 5B), was cloned upstream of a reporterless *lacZ* gene, in an *E. coli* strain that also carried Δ*lacZ* and the *nsoJ138ICR* gene under control of T7 RNA polymerase (Figure 5A). In this strain, IPTG induction leads to production of T7 RNA polymerase, which results in production of CR.NsoJ138I (Figure 4B). If CR.NsoJ138I activates the putative promoter region, β-galactosidase (LacZ) activity will be increased. We carried out two independent experiments to test this. First, IPTG was added to growing cultures with or without the promoter-*lacZ* fusion plasmid, and samples taken over time showed a clear induction (Figure 5C). We also grew cultures under conditions approximating steady-state, where the IPTG (when present) was in the culture medium for at least 10 generations, and the slope of the activity *vs.* culture OD plot is a sensitive measure of expression. As shown in Figure 5D, we observed a 23-fold increase in LacZ activity in response to production of CR.NsoJ138I. This presumably under-represents the actual extent of activation due to combined activation and repression; in the PvuII system, altering the repression-associated C box leads to a huge increase in expression [21]. These results indicate that the fusion is active as a C protein.

## Discussion

### Three classes of RM systems that include R.PvuII orthologs

In attempting to understand the evolution of regulation in C-dependent RM systems, using PvuII as a model, we searched for genes specifying proteins related to R.PvuII. We and others have used this approach to assess the genetic mobility of RM systems [28,47,48], but our purpose here was to examine variation in regulatory mechanisms. Searching for C protein orthologs of unusual size might reveal more fusion proteins, but for our purposes suffers from two problems. First, as REases are highly varied in sequence and structure [33-35], it would be difficult to be certain that the larger C-related proteins were in fact C-REase fusions. Second, as we are interested in regulatory variation, requiring the presence of a C protein would bias the search. In fact, we found three classes of RM systems containing R.PvuII orthologs (Figure 1A): classic PvuII-like systems with independent C and REase proteins, C-REase fused systems such as NsoJ138I, and systems lacking C proteins altogether. We also demonstrated that the C-REase fusion of NsoJ138I was active as both a REase and as a C protein.

### Formation of C-REase fusions

The occurrence of active translational fusions between REases and regulatory proteins has not previously been reported, though automated annotations have indicated the possibility. The standard genetic relationship between C and REase genes should facilitate fusion. Specifically, in the great majority of C protein-dependent RM systems, the C gene is upstream of and in the same orientation as the REase gene; they often overlap (*e.g.*, [49]). In the case of the PvuII-orthologous systems, we expected the C-REase fusions to exhibit REase activity, because R.PvuII tolerates synthetic fusions to yield an active single-chain pseudo-homodimer [50], in which one of the pseudo-monomers has the other fused to its amino end; in addition, R.PvuII with an amino-terminal fusion to maltose-binding protein is active [51]. With respect to carboxyl-terminal fusions to C proteins, it is noteworthy that a structural subclass of these proteins has two additional helices (relative to C.PvuII) at its carboxyl end [52]. We cannot rule out the possibility that, in the native host (*Niabella soli*), some independent expression of the two proteins occurs; however the important point here is that such separate expression is not essential as the fusion protein exhibits both activities.

While C-REase fusions have not previously been characterized, other types of REase fusions have. One class, for example, involves natural and synthetic fusions of the REase and MTase polypeptides [53-56]. This ability to form a variety of active fusions illustrates the remarkable flexibility and modularity of RM systems.

### Implications of C-REase fusions

**Figure 6:**
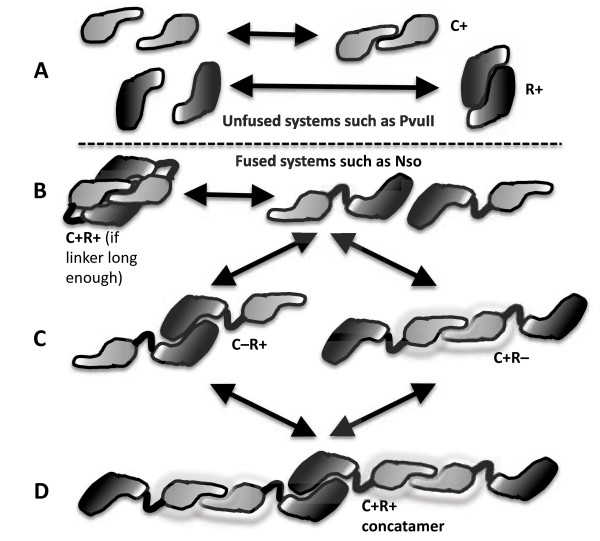
**Possible interactions of C-REase fusion polypeptides. A**. Unfused systems such as PvuII, where the C protein and REase form separate homodimers. **B**. Fused system in which the linker between C and REase regions of the polypeptide is long enough and flexible enough to allow simultaneous dimerization at both C and REase subunit interfaces. **C**. Fused system in which dimerization of the C portion is in competition with dimerization of the REase portion. **D**. Fused system in which concatameric chains can form. See text for details.

The fact that active C-REase fusions can and have formed is intrinsically interesting for what it indicates about the proteins involved. However an equally important question is what (if any) advantage might be conferred by this arrangement. A key difference between C-REase and MTase-REase fusions is that, in the case of C and Type IIP REases, both proteins function as dimers (Figure 6A). Thus MTase-REase fusions are expected to dimerize via the REase portions to yield a dually-active protein, but the C-REase fusions shown in Figure 2 presumably have to dimerize both the C and REase portions to exhibit both activities. This could occur in three ways.

First, the C-associated and REase-associated dimerization interfaces on one fusion polypeptide could both interact at the same time with those on a second fusion molecule (Figure 6B). Symmetry rules would make this dependent on a linker region of sufficient length and flexibility. Looking at the C-REase junction regions in Figure 2, it seems quite unlikely that two molecules of Mru (CR.Mru1279I) or Nso (CR.NsoJ138I) could dimerize both C and REase portions at the same time; for the cyanobacterial fusions this seems less unlikely due to the ~25 additional aa between the two portions.

Second, the two interfaces could dimerize with two different polypeptides, resulting in a concatameric chain (Figure 6D). This second model is not mutually exclusive with the first or third models. It is not clear what benefit chain formation would have, but it is at least theoretically possible at higher protein concentrations, or if the two interfaces have similar *K*d values. For comparison, however, the dimerization interface for R.PvuII is ~2300 Å^2^[25,26], while that for a C protein (C.AhdI) is ~1400 Å^2^[57].

Third, and perhaps most interesting, is that the two portions dimerize competitively (Figure 6C). That is, a pair of fusion polypeptides can form either active REase or active C protein at a given moment, but not both simultaneously. If this competitive dimerization model is true, the results would depend on the relative affinities of the C and REase dimerization interfaces, and would have implications for the relative timing of MTase and REase appearance after the RM system genes enter a new host cell. If the C interface were stronger, this would minimize formation of substantial amounts of active REase early, when low amounts of CR gene transcription were occurring, but increase the sharpness of the induction threshold. On the other hand, if the REase interface were stronger than the C interface (as seems likely given their relative interaction surface areas), there would be early appearance of small amounts of REase activity, but it would take longer for the positive feedback loop to cross the threshold for high expression of the fusion gene, giving more time for protective methylation to occur. Either way, this competitive dimerization model seems to provide the most obvious (and testable) hypotheses of the three interaction modes for possible selective advantages of forming C-REase fusions.

## Conclusions

RM systems closely related to PvuII (as judged by similarity of the REase sequences) have diverse regulatory mechanisms. Most resemble PvuII in having a separate regulatory (C) protein, but we found two that lack C proteins and five in which the C and REase proteins are fused. One of these fusion proteins, from the bacterium *Niabella soli*, is active both as a REase and as a C protein. Fusions between C proteins and REases have not previously been characterized. These results reinforce the evidence for modularity among RM system proteins, and raise important questions about the possible selective advantages of C-REase fusion, including implications of these fusions on RM system expression kinetics.

## Methods

### Strains and cloning

Using the RM system abbreviations listed in the legend to Figure 1 (in alphabetical order), these are the GenBank accession numbers for the DNA sequences: Asp - AJWF01000012.1, Bce - ACCH01000218.1, Esp - AEME01000001.1, Gsp - NC_020051.1, Lba - ACTN01000006.1, Mru - NC_013946, Nso - NZ_AGSA01000028, Oni - NC_019729.1, Pvu - AF305615.1, Pwa - NC_013421.1, Sba - NT_033777.2, Spt - NC_011147.1, Ssp - NC_006511.1, Vei - NC_008786.1, Xsp - AGHZ01000213.1. Initial searching used TBlastN [36]. The Maximum Likelihood method, based on the JTT matrix-based model [58] and with 1000 bootstrap replications, was used to generate a phylogenetic tree (Additional file 1: Figure S2). Evolutionary analyses were conducted in MEGA [59].

The sequence containing the complete R-M system of *Niabella soli* (1837nt, from GenBank accession # NZ_AGSA01000028) was obtained from Genscript Inc. (Piscataway, NJ). Some modifications were made to optimize the distribution of restriction sites, but without changing the specified amino acids. The inferred NsoJS138I C-Box/promoter region (161nt) was also obtained from Genscript, and for cloning purposes XmaI and BamHI restriction sites were placed at the ends. The truncated NsoJS138ICR was cloned into a pACYCDuet-1 vector (Novagen®), with the N-terminus (C protein end) in frame with the His-tag (using BamHI and SaI I sites) and preceded by a T7 promoter, yielding pJL100 (“pNsoShort”). Full length NsoJS138ICR was cloned into this vector, with the C-terminus (REase end) in frame with the His-tag (using the NcoI site and yielding pJL200, “pNso”), initially transforming a strain that already carried the PvuII MTase gene. The synthesized NsoJS138I C-box/promoter region was digested with BamHI and XmaI and ligated into pBH403, which is a derivative of pKK232-8 and contains a promoterless *lacZ* gene between two bidirectional transcription terminators (Paul 2001), yielding pJL300 (“pBoxLac”). The primer pair for making the truncated *nsoJS138ICR* PCR product for pJL100 was 5′-cgtCCATGGacaaaagtcttatgccat and 5′-cgtCCATGGatgaacgaaccaaatgctta, while the primers for the full length product for pJL200 were 5′-aatGTCGACttatttgggattattaatatccttatcac and 5′-aatGGATCCgatgaacgaaccaaatgc.

### REase assays

To assess the enzymatic activity of CR.NsoJS138I, bacteriophage λ DNA (New England Biolabs, Ipswich MA) was used as substrate. The related restriction enzyme PvuII (New England Biolabs) was used for comparison. Assays included 2.36 μg of partially-purified CR.NsoJS138I-His_6_ (see below) or 10 u R.PvuII, with 1.5 μg of λ DNA, and were incubated for 1 h. Temperatures used were 27, 32, 37 and 42°C, in each of four standard reaction buffers (New England Biolabs), and the DNA was resolved on 0.8% agarose gels containing ethidium bromide. pUC19 vector DNA (0.8 μg) was also used as substrate to compare CR.NsoJS138I and R.PvuII. The compositions of NEBuffers 1–4, respectively, are 10 mM Bis-Tris-Propane-HCl, 10 mM MgCl_2_, 1 mM DTT (pH 7.0 at 25°C); 10 mM Tris–HCl, 10 mM MgCl_2_, 50 mM NaCl, 1 mM DTT (pH 7.9 at 25°C); 50 mM Tris–HCl, 10 mM MgCl_2_, 100 mM NaCl, 1 mM DTT (pH 7.9 at 25°C); and 20 mM Tris-acetate, 10 mM magnesium acetate, 50 mM potassium acetate, 1 mM DTT (pH 7.9 at 25°C).

### Inducible expression and western blot analysis

Competent *E. coli* BL21(DE3) cells (Invitrogen, Carlsbad CA), which have isopropythio-β-D-galactoside (IPTG)-inducible T7 RNA polymerase expression, were transformed with pJL100 or pJL200; some of these transformants were made competent and further transformed with pJL300. For protein purification, the QIAexpress® protocol for His-tagged protein purification was followed (QIAGEN, Germantown MD). Overnight cultures were subcultured 1:20 into 250 mL of LB medium at 37°C. IPTG was added to a final concentration of 0.5 mM when the culture reached mid-log phase (OD_600nm_ ~0.46). Cells were grown for another 2.5 h before centrifugation and freezing pellets at −80°C. The QIAexpress® Ni-NTA Fast Start Kit was used to purify 6×His-tagged protein (under naïve condition). The protease inhibitor phenyl-methyl-sulfonylfluoride (PMSF, 0.5 mM) was added to the lysis buffer during the purification of full-length CR.NsoJS138I. Column eluates were immediately transferred into either Diluent B (New England Biolabs recommended storage buffer for R.PvuII; 300 mM NaCl, 10 mM Tris–HCl, 1 mM DTT, 0.1 mM EDTA, 500 μg/ml BSA, 50% Glycerol (pH 7.4@25°C)) or 2× SDS PAGE sample buffer (1:1 solution), and stored at −20°C. Protein concentration was determined by the Pierce 660 nm Assay (Thermo Scientific).

Purified proteins were separated by SDS-PAGE (Novex® 10 ~ 20% Tris-Glycine gradient gel), and were either stained with Coomassie blue or blotted onto PVDF membranes at 30 V for 2 h using an Xcell apparatus (Invitrogen). For signal detection membranes were blocked by incubation at 4°C overnight in 1% BSA-0.1% Tween-20 in phosphate-buffered saline, followed by incubation with a 1:1,000 dilution of mouse anti-His-tag monoclonal antibody (EMD Millipore, Billerica MA) for 2 h at 4°C, followed by three 10-min washes. The blots were then incubated for 2 h with 1:15,000 horseradish peroxidase (HRP)-conjugated goat anti-mouse IgG (Invitrogen) at room temperature. After three 10-min washes, protein bands were visualized by ECL Plus enhanced chemiluminescence (GE Healthcare Biosciences, Piscataway NJ) and image captured using an Alpha Innotech FluorChem HD Imaging System using either white light or dual 302/365 Å illumination. Adjustments of brightness and contrast were carried out to better visualize data, but in all cases the changes were applied to the complete image. The pre-stained MW markers used were SeeBluePlus (Invitrogen).

### Assays for C protein activity

Plasmid pJL300 was used to transform *E. coli* BL21(DE3) carrying pJL200 (plasmids described above). The β-galactosidase (LacZ) assay was based on hydrolysis of O-nitrophenyl-β-D-thiogalactoside (ONPG) [60]. Briefly, activity and culture density were measured at 20–30 min intervals during exponential growth. The units for this assay were calculated by dividing the measured A_420nm_ (released nitrophenol) by the time allowed for the reaction and volume of permeabilized cells used for the reaction. For plots *vs.* time, culture density (OD_600nm_) was also in the denominator, yielding standard Miller units. For plots *vs.* culture density, this term was omitted from the denominator, yielding modified Miller units (1000 × ΔA_420nm_ min-1 ml-1). Specific activity was obtained by determining the slope of a plot of LacZ activity versus the culture density via linear regression.

## Abbreviations

MTase: Modification DNA methyltransferase; REase: Restriction endonuclease; RM: Restriction-modification; LacZ: β-galactosidase; IPTG: Isopropythio-β-D-galactoside; ONPG: O-nitrophenyl-β-D-thiogalactoside; HRP: Horseradish peroxidase; PMSF: Phenyl-methyl-sulfonylfluoride; OD: Optical density; SDS: Sodium dodecylsulfate.

## Competing interests

The authors declare no competing interests.

## Authors’ contributions

RB conceived the study; RB and JL carried out the sequence analyses and designed experiments; JL performed experiments; RB and JL wrote and approved the manuscript. Both authors read and approved the final manuscript.

## Supplementary Material

Additional file 1: Figure S1 Alignment of R.PvuII orthologs. **Figure S2**. Phylogenetic analysis of R.PvuII orthologs. **Figure S3**. Confirmation of specific digestion conditions. **Figure S4**. Effect of enzyme dilution in three reaction buffers. **Figure S5**. Test of CR fusion protein production. Click here for file
