# Supplementary material for: Naturally-occurring, dually-functional fusions between restriction endonucleases and regulatory proteins
Source: BMC Evol Biol. 2013 Oct 2;13:218. doi: 10.1186/1471-2148-13-218 (PMC3850674; doi:10.1186/1471-2148-13-218)

Additional File with Supplementary Information for

Naturally-Occurring, Fully-Functional Fusions Between Restriction Endonucleases and Regulatory Proteins

Jixiao Liang[1] and Robert M. Blumenthal[1,2*]

*Correspondence: Robert.Blumenthal@utoledo.edu
[1]Department of Medical Microbiology & Immunology, College of Medicine and Life Sciences, University of Toledo, 3100 Transverse Drive, Toledo, OH 43614, USA
[2]Program in Bioinformatics, University of Toledo, 3100 Transverse Drive, Toledo, OH 43614, USA

BMC Evolutionary Biology

Figure S1. Alignment of R.PvuII orthologs. These sequences are from the set of REases orthologous to R.PvuII, that was used to identify RM systems related to the PvuII system. Positions are shaded where ≥80% (12/15) of these REases have the same amino acid, and bolded positions refer to functionally-defined amino acids as indicated in Figure 2. RM abbreviations are listed in the legend to Figure 1, but “Pvu” is the original search seed R.PvuII. The abbreviations indicated in bold were found to have C-REase fusions (though only the REase portion is shown here).

```
Pvu           MSHPDLNKLLELWPHIQEYQDLALKH-GINDIFQDNGGKLLQVL-LITGLTVLPGREGNDA
Asp           …RRSDLERLQTALGAIQELQSLANEY-GINDVFQDNGGKVLQVL-ILLGLRISPGREGNDA
Bce           -------KLNNLFPHIREYQKLASELYSINDVFQDNGGKLLQVLMITGLQNLDGSREGNDA
Esp         MKFELHQDWSNLIALWPQVEEYQRLANKH-GINDIFQDNGGKLLQVL-LLLSLKVLPGREGNDA
Gsp           …RRSDLERLQTALEAIQELQTLANEY-GINDVFQDNGGKVLQVL-ILLGLRISPGCEGNDA
Lba     MSFYDEKHRAEIAKIEQLWRNLNELDQ-MAHAY-GISDIFQDNGAKVLQQL-IYLNMSILPGREGNDC
Mru          …----DIAILEALFPYIRRYQILASRY-GIADIFQDNGGKLLEIL-IRTGLKIVPGREGNDA
Nso           …KNEELIALNMILPQIRRFQTLAEAN-GINDIFQDNGGKLLQVL-LHTGLKILPGREGNDA
Oni           …RISDAKRLENAMKSIQELQNLASEY-GIADIFQDNGGKTLQLL-ILLGLRISPGREGNDA
Pwa         MKFELHSDWQKLIDLWPQVEEYQRLANKH-GVYDIFQDNGGKLLQVL-LLLSLKVLPGREGNDA
Sba         MKFELHQDWSNLIALWPQVEEYQRLANKH-GINDIFQDNGGKLLQVL-LLLSLKVLPGREGNDA
Spt         MKFELHQDWSNLIALWPQVEEHQRLANKH-GINDIFQDNGGKLLQVL-LLLSLKVLPGREGNDA
Ssp      MAFFDNEHKKEIERIEGLWAKLEELRDFAKQY-GIDDIFQDNGAKVVQQL-IYLYLNALPGREGNDA
Vei     MTQANHSAEETDKQKFERLFPCIREYQVLASKY-KINDIFQDNGGKYLQLL-MILGLTTDGTREGNDA
Xsp         MTLKPHQDYERLQAAWPSVEEYQKLATKH-GIDDIFQDNGGKLLQVL-LLLGLRIIPGREGNDA

Pvu VDNAGQEYELKSINIDLTKG---FSTHHHMNPVIIAKYRQVP-WIFAIYRGIAIEAIYRLEPKDLEFYYDK
Asp IDAEKNEYELKTINKLLSKS---VTTNHHLNLDILAKYRAVKAWYIAVYEGILLKAIYRVDPTSLETKFSY
Bce VDSEGNEYELKSVNINLTQS---FSTNHHLNQHIIDKYRKVD-WIFAVYKDIELVEIYKMTPEMIEPYYTK
Esp VDVTGTEFELKSVNVELTKS---FSTHHHMNPTIIAKYRQVP-WVFAIYSNITIRSVYLLMPGDLEVFYDK
Gsp IDAEGHKYELKTINKLLSRS---VTTHHHLNLDILAKYRAVKAWYIAVYEGIVLKAIYRVAPTSLESKFSY
Lba ISTSGTEWEMKSINLETSAS--GFSTNHHTTHDIIAKYRQVP-WTFAIYYGINLAEMYVMTPEMLEPLYQH
Mru VDEQGQEYEIKSVNVELTRS---FSTHHHLNPSILDKYRRVR-WIFAVYRNIELQEIYCLEPQQLERFFSD
Nso VDDAGNEYELKSVNIRLTKS---FSTHHHMNPTIIKKYRKVD-WIFAVYEGIELLEIYKLKPAHLEPYYVA
Oni IDAEGKEYELKTINIALNRS-GGVTTHHHLNEIILEKYRRVEAWYIGLYEGITLKEIYKLTPEMLEPKFKE
Pwa QDITGTEFELKSVNVELTKS---FSTHHHMNPSIISKYRKV-PWIFAIYSNITIRSVYLLMPIELEFFYEK
Sba VDVTGTEFELKSVNVELTKS---FSTHHHMNPTIIAKYRQV-PWVFAIYSNITIRSVYLLMPDDLEVFYDK
Spt VDVTGTEFELKSVNVELTKS---FSTHHHMNPTIIAKYRQV-PWVFAIYSNITIRSVYLLMPDDLEVFYDK
Ssp TSRRNPTIEWEMKSINLDTTASGFSTNHHLNTEILQKYRRV-PWSFAIYHGITLEAIYVMNARDLEPYFQK
Vei VDSFGNEYEIKTVNLELQHQ---FTTHHHMNPAIIAKYRKV-DWYFAAFKNIELQVIYRLKPDHMESFYAR
Xsp VDANGREYELKSVNIELVKG---FSTHHHMNPAIIAKYRQV-PWIFAIYRHIALQAVYLLEPTDLEFYFQK

Pvu WERKWYSDGHKDINNPKIPVKYVMEHGTKIY--*
Asp WESRIR-GGMESINNPKIPLSLIEKEGELVY--*
Bce WETQYK-TTGKDINNPKIPLKFVRENGIEIYKNKEGADFFYAPIINDKEHIELNKIKQGELF*
Esp WERQWHERDGRDINNPKIPVKYVIENGKLLWTPEIEEQIDLGGFEADA*
Gsp WESRIF-EGMKSINNPKIPLALIEKEGKLVY--*
Lba WEAKLQ--TMTHLNNPKIPVKYVREHGIKVFPINPYNPIDPDSIN*
Mru WERKWRAQGGKDINNPKIPLRYVQREGRLIYSH*
Nso WEKKWSADGDKDINNPKIPLKYVRLHGIRLL--*
Oni WEEKVR-LRNEPLNNPKIPLKLVKK-GQLVYSH*
Pwa WEGQWHERGGRDINNPKIPVKYVMEHGCLLWSNTSPQELLWTPEITKQGDLGGFEAEN**
Sba WERQWYERDGKDIQS*
Spt WERQWYERDGKDINNPKIPVKYVIEYGKLLWSNATPEELLWTPEIEEQIDLGGFEAEN**
Ssp WEEKLR--TQKDINNPKINVNFVRENGTMIYPIHEENPIDPDSIFD*
Vei WEKKWHDDGGKDINNPKIPLAHVMEYGEIIWLPEGSAGFVRPKPGPNRLAKARRKKSP*
Xsp WEGKWHADGGKDINNPKIPAKYVLKHGRLIHGQAPDLSTRRRVQPTPDDAAGFEPDEV*
```

Figure S2. Phylogenetic analysis of R.PvuII orthologs. The evolutionary history of the sequences shown in Figure S1 was inferred by using the Maximum Likelihood method based on the JTT matrix-based model. The tree with the highest log likelihood (-2452.1787) is shown. The percentage of trees in which the associated taxa clustered together, following 1000 bootstrap replications, is shown next to the branches. Initial tree(s) for the heuristic search were obtained automatically as follows. When the number of common sites was < 100 or less than one fourth of the total number of sites, the maximum parsimony method was used; otherwise BIONJ method with MCL distance matrix was used. The tree is drawn to scale, with branch lengths measured in the number of substitutions per site. All positions containing gaps and missing data were eliminated. There were a total of 132 positions in the final dataset. Numbers at branchpoints indicate the percentage of trees, from the 1000 bootstraps, that included that branching.

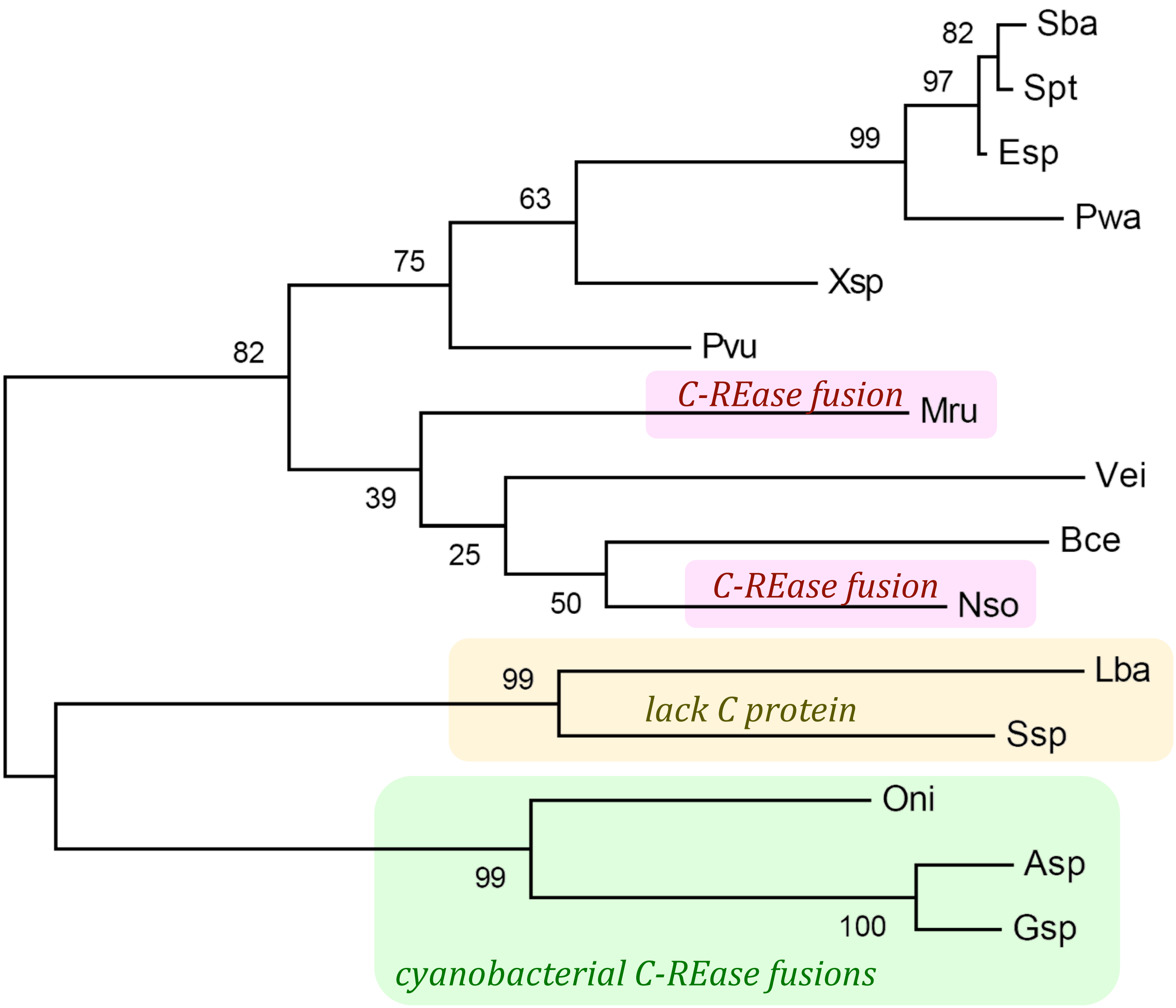

Figure S3. Confirmation of specific digestion conditions. In some buffers, R.PvuII or CR.NsoJS138I was active or inactive at a single temperature (as shown in Figure 4). To confirm this temperature specificity, those two buffer-enzyme combinations were re-tested using 2 µg (upper lanes) or 1.5 µg (lower lanes) of bacteriophage λ DNA. M = size markers; U = uncut DNA. The image is a UV-illuminated agarose gel containing ethidium bromide.

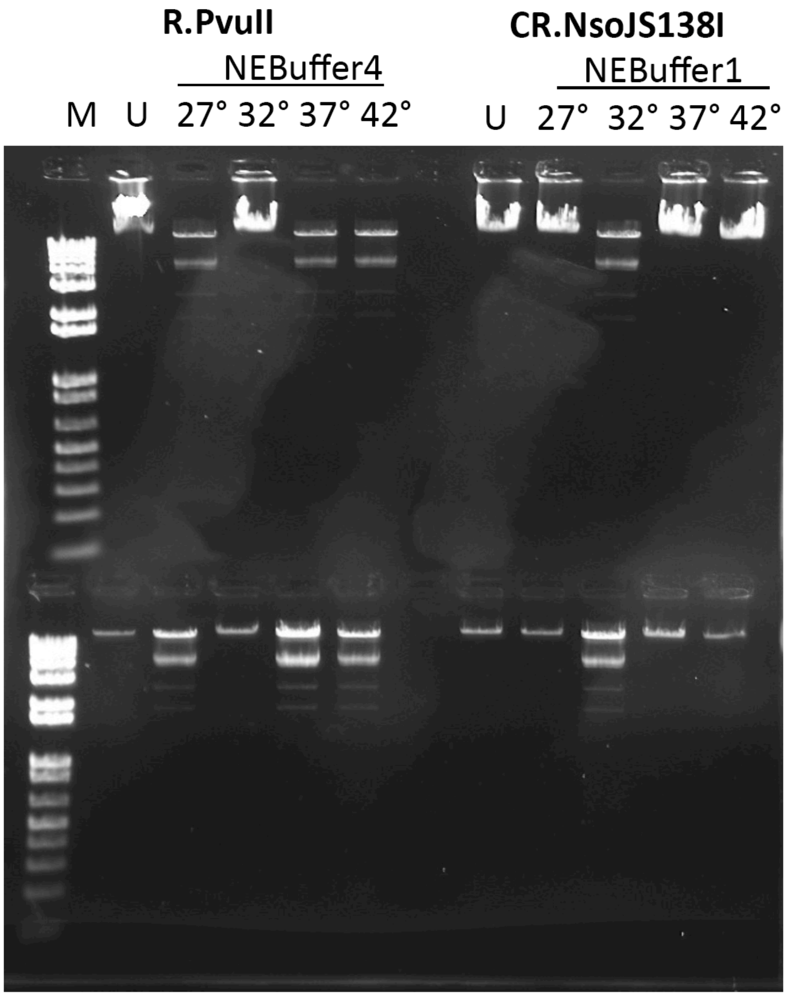

Figure S4. Effect of enzyme dilution in three reaction buffers. Choosing conditions in which both R.PvuII and CR.NsoJS138I were active (Figure 4) – 32 °C in NEBuffers 1, 2, or 3 – digestions of of bacteriophage λ DNA were set up with twofold serially-diluted enzyme (from undiluted to 1/32, where undiluted is the amount used to generate Figures 4 and S3). The diluent was NEB diluent B (see Methods). The image is a UV-illuminated agarose gel containing ethidium bromide.

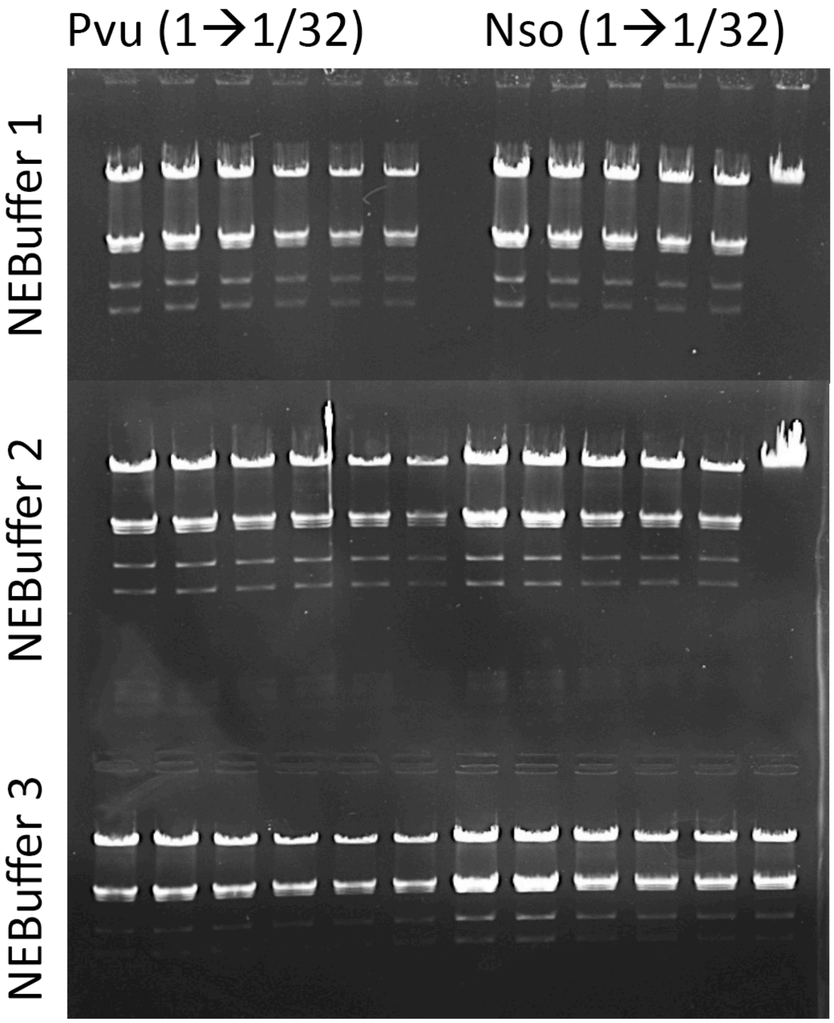

Figure S5. Test of CR fusion protein production. Lanes, from left included 4.5 µg protein, MW markers, lysis buffer + PMSF control, and 5.5 µg protein. Production of NsoJS138I C-REase fusion protein, with an amino-terminal His_6 tag, was induced using a T7 RNA polymerase-dependent promoter (see Methods). The clone had a small carboxyl-terminal deletion (in case the REase activity proved to be toxic). Centrifugally-clarified whole-cell extracts, containing protease inhibitor PMSF, were passed over affinity columns and resolved on a 10-20% gradient acrylamide SDS gels. The gel was blotted to PVDF, blocked, and probed with HRP-conjugated anti-His tag antibodies. The image on the left was detected using lights with 365/302 nm dual-wavelength for the visibility of markers; the image on the right is from chemiluminescence alone. For details see Methods and the legend to Figure 3.

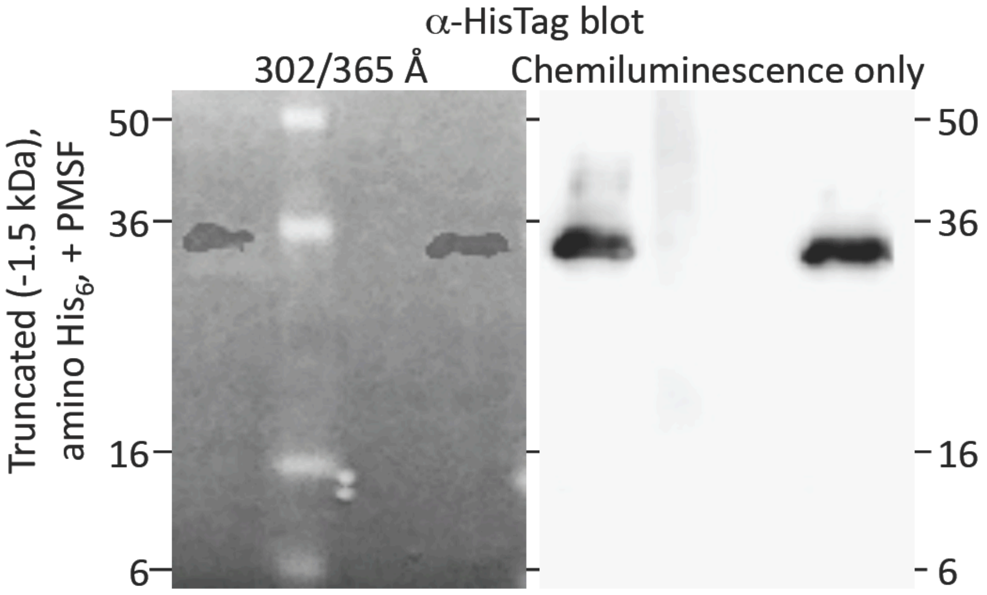

Supplement: Additional file 1: Figure S1 — Alignment of R.PvuII orthologs. Figure S2. Phylogenetic analysis of R.PvuII orthologs. Figure S3. Confirmation of specific digestion conditions. Figure S4. Effect of enzyme dilution in three reaction buffers. Figure S5. Test of CR fusion protein production. [file 1471-2148-13-218-S1.pdf]
